# When Significant Others Turn into Ambivalent Others: An Intersubjective-Phenomenological Perspective on the Generation of Internal Conflicts

**DOI:** 10.17505/jpor.2025.28327

**Published:** 2025-10-02

**Authors:** Igor Krasilnikov

**Affiliations:** The Poltava Regional Clinical Medical Cardiology Center, Poltava, Ukraine

**Keywords:** internal conflict, ambivalence, significant others, ambivalent others, life world, phenomenology of intersubjectivity, inter-subjective-phenomenological uncertainty, phenomenological presence of the other

## Abstract

Internal conflicts are typically seen from an *intra*personal perspective in the literature, both as to how they are generated (e.g., by focusing on internal motives) and how they are to be solved (e.g., by emphasizing the therapeutic role of insight and cognitive processing). The present paper argues for an *inter*personal perspective, where internal conflicts are seen to develop when the experience of significant others is transformed into an experience of ambivalent others. It is hypothesized that the inter-affective connection of the life worlds between a person and a significant other is of decisive importance in understanding the process of formation of the person’s internal conflict. In this model, which is based on an intersubjective phenomenological paradigm, the affective internal conflict of the self is understood as based on the phenomenological presence of the ambivalent other in the subject’s life world. It is argued that this model of internal conflicts can have theoretical and practical significance for psychological counseling and psychotherapy.

## Introduction

The purpose of this paper is to present a new model of the development of internal conflicts that can have theoretical and practical significance for the understanding of psychological health and psychopathology, and for psychological counseling and psychotherapy. According to this model, which is grounded in an intersubjective-phenomenological paradigm, internal conflicts are seen to develop when the experience of significant Others turn into the experience of ambivalent Others. The paper contains five main sections. The first four sections section are devoted to four concepts that are of primary importance for the present model: *internal conflicts, ambivalence, significant Others,* and the *intersubjective-phenomenological paradigm*. The first section contains a brief review of the existing literature on internal conflicts, whereas the second section reviews some recent research on ambivalence. The third section is about the concept of significant Others, and some related concepts, whereas the fourth section provides a brief discussion of the intersubjective-phenomenological paradigm. In the final fifth section of the paper, we develop our model of internal conflicts as generated in the emotional transformation of the experience of significant others into an experience of ambivalent others.

## Traditional Perspectives on Internal Conflicts

In a general philosophical sense, internal conflicts are typically defined in terms of internal contradictions or internal disharmony, which manifest themselves in ”a struggle of motives”, and the need for a personal choice among one of several mutually exclusive alternatives. In psychology, a more precise definition is to consider an internal conflict as an emotionally significant state of the individual, that requires a personal decision (personal choice) in a situation where it is impossible to simultaneously satisfy various vital needs (Antsupov & Shipilov, 2004; Grishina, 2008). Among the limitations of such a definition is that it does not answer the question of how the formation and resolution of an internal conflict occurs.

Four important theoretical approaches to the understanding of internal conflicts are the psychoanalytic, the social-cognitive, the existential-humanistic, and the activity-based approach (for a detailed and systematic description of these models, see Krasilnikov, 2014). In the classical psychoanalytical model (e.g., Fenichel, 1995; Freud, 1966), internal conflicts are seen to arise between the deep structures of the Id and the Super-Ego of the unconscious mental sphere, and psychological defenses play a leading role. In social psychoanalysis (e.g., Adler, 1923; Fromm, 1994; Horney, 1945), the internal conflict is more connected to the individual’s socially conditioned motives, but the focus is still on intrapersonal processes. Similarly, in the social-cognitive tradition, internal conflicts have been defined in terms of “cognitive dissonance” (Festinger, 1957) or introjected socialcognitive schemes and fixed internal irrational attitudes (e.g., Beck et al., 1979; Ellis & Dryden, 1997). Theories within the existential-humanistic tradition (e.g., Binsvanger, 1963; Boss, 1979; Bugenthal, 1976; Frankl, 2014; Laing, 1961; May, 1994; Yalom, 1980) define internal conflict in terms of deep, irremovable self-reflective anxiety, a feeling of loss of sense of life, and the authenticity of the self. Finally, in the activity-based approach (e.g., Leontiev, 1978; Vygotsky, 1978), internal conflict is considered as a discrepancy in the need-motivational sphere of the individual, between various needs, motives, goals and ways of achieving them. The resolve of inner conflict is seen to require deep self-reflection on the activities, goals, needs, etc., that are in conflict.

Typical of these approaches is that they understand internal conflict primarily as an internal state of ambivalence in the individual person. This is especially clearly manifested in psychotherapeutic practices where the main psychological mechanism for resolving internal conflict is usually considered to be deep self-reflection (insight) or cognitive psychocorrection. One overlooked aspect is that, even when the internal conflict is sometimes recognized as having interpersonal roots, the resolution of internal conflicts is still seen as a matter of intra-psychological processes.

## The Concept of Ambivalence

As described above, traditional approaches typically understand internal conflicts as internal states of ambivalence in the individual person. As a first step beyond these intrapsychological approaches, it is important to consider the understanding and typology of internal conflicts in the vectorfield theory of Kurt Lewin.

### Ambivalence as internal conflict in Lewin’s vector-field psychology

A significant step was taken by the social psychologist Kurt Lewin (1936) in his concept of vector-field behavior, where he introduced the concept of *life space*. According to Lewin, an individual’s personality is emotionally integrated into the life space (field), and behavior is under the powerful influence of motivational and value forces from specific objects (subjects) in this field. At the same time, the vector direction of the individual’s behavior is determined by their perception of the life situation, which reflects both the internal qualities of the personality and the affective characteristics of the surrounding social environment. Behavior is determined by the person’s internal intentional orientation to specific affective ”objects” of the life space.

Lewin developed a typological model of internal conflict, where he identified three types of internal conflicts, based on the ratio of motivational-valence forces (attraction or repulsion) directed at specific social objects of the field. The first type of conflict, *attraction-attraction*, consists in the need to choose between two equally positive-affective external objects. The second type is *repulsion-repulsion*, where the choice must be made between two negative-affective objects. The third type is *attraction-repulsion*, where the choice is associated with one object, in relation to which the individual experiences ambivalent motivation.

In fact, however, the first two types of internal conflicts can also be considered as subtypes of ambivalent internal conflict. This can be explained as follows. The very fact of the need for a subjective choice of the first or second social object indicates that the subject falls into the field of attraction to both objects. The presence of a second social object through mental comparison activates in the subject’s consciousness those affective ”relatively attractive” qualities that the first object does not have: and vice versa with relatively ”unattractive qualities”. This is precisely why subjective difficulty in choosing arises; in essence, a dual affective-ambivalent attitude toward each of the two objects is experienced. Based on this, we believe that the ambivalent motivation of the individual in relation to each social object localized in the life space is the central characteristic of any type of internal conflict. Now let us move on to the consideration of modern psychological studies analyzing the ambivalence of the individual as an internal conflict in various aspects.

### Theories and empirical research on ambivalence

Although the research literature on ambivalence has grown considerably during the last few decades, it is not easy to summarize, as it spreads in many different directions. Some of these studies address conflicting motives and feelings when a person has make a choice, such as for example whether to express or suppress one’s emotions in relation to other persons (Glazer, 2021), whether to engage or not to engage actively in psychotherapy (“resistant ambivalence” e.g., Engle & Arkowitz, 2006), whether to attend cardiac rehabilitation if you are a cardiac patient (“patient ambivalence”; Everett et al, 2009), and adolescents’ decisional ambivalence about having intercourse (Pinquart, 2010). Other studies focus on ambivalent attitudes towards various phenomena in the surrounding world (e.g., abortion, organ donation, euthanasia, contraception, minority groups, tobacco, etc.; Schneider & Schwarz, 2017), or physical exercise (Sparks et al., 2004).

But several studies also focus on ambivalence in interpersonal contexts. For example, there are studies which show that ambivalent feelings towards others are associated with negative effects on health, such as elevated blood pressure (Holt-Lunstad et al., 2003) and coronary-artery calcification (Uchino et al., 2014). On the other hand, there are also studies which report that higher implicit ambivalence among newlywed couples can be associated with stronger motivation to make efforts to solve current marital problems (Faure et al., 2022).

Different theorists and researchers have discussed possible positive versus negative consequences of ambivalence. Based on a review of research on ambivalence in organizational contexts, Rothman (2017) concludes that ambivalence is ubiquitous in organizations, and that although ambivalence has traditionally been seen as a source of distress, there is also evidence that ambivalence can sometimes be beneficial. As they conclude,

the myriad negative and positive outcomes of ambivalence may be organized around two key dimensions that underlie most research on the effects of ambivalence: (1) a flexibility dimension: inflexibility to flexibility and (2) an engagement dimension: disengagement to engagement. (Rothman et al., 2017, p. 33)

This would mean that ambivalence, at least under some conditions, can be associated with increased flexibility and engagement. Hartmann and Zimberoff (2004), somewhat similarly, maintain that the opposite of ambivalence is a rigid intolerance for ambiguity, nuance or paradox, and that the synthesis of the two is a “passionate commitment in the face of ambiguity” (p. 3). Razinsky (2017) similarly speaks about a form of conscious ambivalence, which can be expressed in creative compromise actions.

On the other hand, it might be argued that strong forms of ambivalence are more difficult to use creatively. Blumenthal-Barby (2021), for example, differentiates between *residual* ambivalence and *paralyzing* ambivalence. In residual ambivalence there is a conflict about what to do, but it is possible to solve the conflict. In paralyzing ambivalence, on the contrary, no solution can be found, which leads to a stuck form of ambivalence, where self-management is lost. Albertson et al. (2015) believe that the concept of ambivalence is used too broadly and should be limited to cases of strong internal conflict.

Several authors emphasize that ambivalence is part of the human condition. The sociologist Bauman (1993) sees a source of ambivalence in the postmodern fragile world, with its ambiguity and uncertainty, which gives rise to ambivalent feelings; however, he also suggests that instead of trying to eliminate ambivalence, we should learn to acknowledge and manage it. Weisbrode (2012) suggests that ambivalence is inherent in the psychological nature of man and is a rather complex state of conflicting human experiences that cannot be resolved by merging opposing aspirations; there is no simple ”golden mean”. However, ambivalence forces a person to think more deeply and analyze their life situation.

More specific examples are found in the literature on psychotherapy. In Miller & Rollnick’s (2023) method of motivational interviewing, for example, ambivalence is considered as a dynamic emotional state of readiness for po-sitive changes. In this method, the client’s ambivalence is considered to be a normal stage of psychological growth of awareness and is very important for overcoming addictive forms of behavior. In motivational interviewing, the reflection of the ”two minds” is activated, which helps to realize those negative internal resistances that ”protect” addictive forms of behavior. According to the authors, the reflexivity of polyphonic self-understanding and the ”two chairs” technique allow for a deeper understanding and overcoming of the client’s self-destructive ambivalent behavior.

## The Concept of “Significant Others”

The concept of *significant Other* emphasizes the significance of other people in a person’s life. This is a concept that appeared first in the works of Sullivan (1953) as part of his views on how disturbed interpersonal relationships lead to psychopathological manifestations. An emphasis on significant others is also found in Klerman and Weissman’s shortterm interpersonal therapy (Klerman et al., 1984), which is directly influenced by Sullivan’s thinking, and in the writings of some psychoanalysts (e.g., Kernberg, 1984).

A partly similar concept is that of *self-object* as developed by Kohut (1984), who speaks of relations not between Self and *Others*, but of relations between the self and *self-objects*. A self-object, in Kohut’s theory, is another person who is emotionally important for the person’s self-experience; this may be because the other person provides empathic understanding, or serves as a safe role model, or functions as an “equal” to identify with. Partly similar concepts are also found in Bowlby’s (1988) attachment theory, which points to parents’ role as serving as a *secure base* for a child to return to for support after exploration, and as a *safe haven* that can provide comfort, reassurance, and protection when the child experiences distress or fear.

Empirical research has documented different ways in which significant others play a role in people’s lifeworld. Andersen et al. (2002), for example, studied how memories of previous experiences with significant others remain in the form of mental representations, that can be activated by new interpersonal interactions and then influence the processes of self-regulation and the sense of vulnerability positively or negatively; they describe these processes in terms of the psychoanalytical concept of *transference*. Yet another perspective on the influence of significant others has been presented by Aron et al. (2004), who argue that we tend to include significant others into our Self. They describe this as a tendency for “self-expansion”, in the sense that we include the resources, perspectives, and identities of close others as our own. A third example of the role played by significant others is exemplified in the research by Poerio et al. (2016), which showed that even imaginative fantasies about a significant other can help to overcome feelings of loneliness and improve emotional well-being. There are also studies that appeal to the concept of ‘significant others’ in the context of supportive and helping behavior, for example, for patients with medical diseases such as rheumatoid arthritis (Bergström et al., 2020)

## The Intersubjective-Phenomenological Paradigm

Lewin’s concept of *life space*, as described above, is partly reminiscent of the phenomenological concept of *life world* (Husserl, 1970). When Husserl introduced the concept of ‘lifeworld’, he was referring to the fact that we all live in an everyday world where we are engaged in various practical, goal-oriented concerns and efforts, which give meaning to our existence. People’s actions and experiences are always directed towards one thing or another, either as an end or as a means, but always based on some kind of practical interest – work, family, leisure activities, studies, research, etc. The lifeworld includes both material objects (such as houses, chairs and tables) and other people and social relationships (family, work, associational involvement, etc.). It is important to note that, even though the concept of lifeworld primarily refers to the interpersonal, collective everyday world that we share with other people, each individual has their own personal version of this lifeworld. In this process, we are by no means isolated individuals, but are in contact with other human beings, in a way that means that each of us can take part in each other’s lives. The lifeworld is thus an *inter-subjective* world.

An essential part of a person’s life world is the presence of other persons and the relations between Self and Others. The *intersubjective-phenomenological paradigm*, as defined here, is a research approach that focuses on people’s experience of their relations between Self and Others, and the interconnections between the overlapping life-worlds of different individuals. One example is Fuchs and Koch’s (2014) model, according to which encounters between self and other involve two cycles of embodied *inter-affectivity*, with a circular interplay of expressions and reactions, where the emotions of each partner constantly modify the other’s emotional state. In more general terms, Fuchs (2018) describes an embodied and ecological view of the mind, where the nonverbal interaction between group members is seen as key to the dynamics of their interrelations. The interpersonal interaction is conceptualized as an ongoing process of interbodily resonance, mediated by the participants’ reciprocal expressions and impressions.

Pollio (1997) notes that the phenomenological tradition tries to overcome the idea of classical psychoanalysis, namely, the consideration of everything important about other people as being inside oneself. Ideas related to the intersubjective-phenomenological approach, however, have also begun to appear within psychoanalysis, where some authors (e.g., Atwood & Stolorow, 2014) consider the intersubjective approach (mainly in the psychoanalytic situation) as phenomenological. They also believe that the intersubjective context plays an exceptional role in the pathogenesis of all forms of psychopathology. Similarly, within Gestalt psychotherapy, Wheeler (2015) speaks about the limitations of the methodology of individualism and the need for a methodological turn to a phenomenology of intersubjectivity.

## An Interpersonal Model of Internal Conflicts

**Figure 1:**
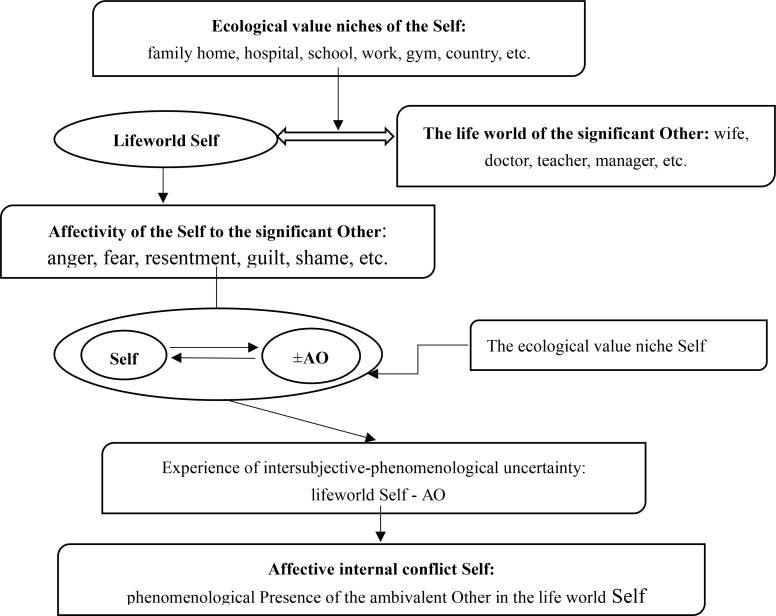
Structural components and procedurality of the formation of an affective internal conflict of the Self

Our hypothesis is that internal conflicts arise from a person’s relations with significant others (e.g., parents, spouses, neighbors, boss, doctor, politicians, etc.), who can emotionally and value-wise influence that person’s way of existence and feeling of life. Figure 1 describes the model in terms of its main structural components and the procedural nature of the formation of an affective internal conflict. As seen at the top of the figure, a person’s lifeworld is conceptualized as containing different ecological niches (family, school, work, hospital, etc.) that contain significant others, and relations that involve encounters between the lifeworld of the Self and the life-worlds of these Others. In the interaction between the Self and the significant Other, an inter-affective and communicative connection arises, and an intersubjective interpenetration of life worlds is realized. The person, consciously or unconsciously, accidentally or purposefully, lets the Other into their life world, and this significant Other becomes an important and inseparable part of the phenomenological life world of the Self. For various reasons, however, different kinds of relational disturbances may arise between the Self and the significant Other, which leads the person to respond with various affects (e.g., anger, fear, resentment, guilt, shame, etc.) to the Other. When this occurs, the significant other is transformed into an ambivalent other (AO), which leads to experiences of intersubjective uncertainty, and eventually into the experience of an internal conflict.

Basically, what makes other persons into significant Others is that they are connected with the most important vital meanings of the existence of the Self. In other words, the Self is dependent on the actions of the Significant Other for his/her ways of existence. This differs from one niche to another. Thus, for a child, the significant Other may be the mother, and for a patient it may be the doctor. Explicitly or implicitly, the significant Other is existentially-phenomenologically present in the life world of the Self; although all significant others are existentially valuable and necessary for the subject in certain life circumstances, this may not always be explicitly recognized by the subject.

Being in a common ecological niche, such a socio-affective and communicative connection arises between the Self and a significant Other (the Self and a neighbor, the Self and a doctor, the Self and a teacher, the Self and a colleague, etc.) that even in any course of life events, it is not always possible to break this connection by a single volitional effort on the part of the subject. Thus, the presence of a significant Other in a specific ecological niche of a person’s life world means that this significant Other exerts a powerful affective-motivational force of connection and attraction on the Self.

On the other hand, due to various life circumstances, various kinds of relationship disturbances may arise between the Self and the significant Other: frustrations, conflicts, disagreements in the assessments of certain events, failure to fulfill mutual expectations, etc. Even a threat to the life of the significant Other may induce anxiety in the subject. In this regard, the phenomenology of the emotional experiences of the Self in the intersubjective context changes. In the phenomenology of the affectivity of the Self, a complex of negative emotional experiences appears, such as for example, fear of a significant Other (when the Other is capable of causing a feeling of personal danger), anxiety for a significant Other (when the Other is in danger himself), aggression towards a significant Other, resentment, guilt, shame, wounded pride, etc. Thus, a doctor with his careless communication can cause iatrogenic, hyper-anxious states in a patient; a friend can offend or betray at an unexpected moment; a son can commit a criminal offense with his thoughtless actions, etc. In certain life circumstances, the subject discovers qualities of a significant Other that are unacceptable to him/herself and thus finds himself in a difficult life world.

In such situations, an emotionally negative intentionality of the Self in relation to the significant Other is formed. This allows us to say that the significant Other evokes a motivational force of repulsion in the Self’s feelings. At the same time, the emotional significance of the Other may not disappear from the subject's experiences. The Other as a significant personality may retain its force of attraction, but now this Other begins to evoke an opposite force of repulsion in the Self's feelings. Thus, a psychological transformation of the significant Other into an ambivalent Other occurs: the Other begins to evoke an ambivalent affectivity in the feelings of the Self, the simultaneous presence of affective forces of attraction and repulsion.

The ambivalent Other, with its affective contradictoriness, evokes in the subject experiences that we designate as experiences of *intersubjective-phenomenological uncertainty*. Here the subject does not always realize and deeply understand what is happening in his relations with the ambivalent Other, and it is difficult for the subject to be self-determined and make a decision about certain real actions in relation to the ambivalent Other. The subject in the intersubjective context faces personal questions to which it is sometimes difficult to find quick answers and solutions. “Why did this Other act this way towards me?” “How should I relate to this Other and what can I expect from him?” “What actions should I take towards this Other in order to eliminate my personal emotional suffering?” The entire phenomenology of the experiences of the Self essentially depends on the complex of affectivity that connects the subject with the ambivalent Other.

We can single out and speak about the processual formation of the affective internal conflict of the Self and understanding it as the phenomenological presence of the ambivalent Other in the life world of the subject. This means that the formation of the affective internal conflict of the Self is carried out on the basis of the psychological transformation of the significant Other into an ambivalent Other in the phenomenological life world of the Self.

## Conclusion

Internal conflicts have traditionally been seen from an intrapersonal perspective both as to how they are generated (e.g., by focusing on internal motives) and how they are to be solved (e.g., by emphasizing the therapeutic role of insight and cognitive processing). The present paper argues for an interpersonal perspective, where internal conflicts are seen to develop when the experience of significant others is transformed into an experience of ambivalent others. We suggest that this model of internal conflicts can have theoretical and practical significance for psychological counseling and psychotherapy.

More generally, we suggest that the intersubjective-phenomenological paradigm, as described above, has significant theoretical explanatory potential. In this model, the formation of the internal conflict of the Self is based on the emotional transformation of a significant Other into an ambivalent Other in the life world of the subject. Ambivalent experiences in relation to a significant Other, representing an internal conflict of the Self at the personal level, can permeate various spheres of human activity. Our hypothesis is that failure to resolve such conflicts creates high risks of developing personality deviations, psychopathological and psychosomatic disorders. The suggested model also affords a conceptual expansion of ideas about psychotherapeutic and auto-psychotherapeutic strategies for resolving affective internal conflicts of the Self, based on the need to transfer the intrapsychic activity of the subject to an interpersonal (intersubjective) contextuality. The study of how such a psychological process can be developed, however, requires additional conceptual exploration. To summarize, our hypothesis is that the present model allows for a deeper conceptual understanding of the intersubjective-phenomenological determinants underlying the formation of internal conflicts of the Self and the development of appropriate psychotherapeutic strategies.

## Action editor

Lars-Gunnar Lundh served as action editor for this article.

## Open access

This article is distributed under the terms of the Creative Commons Attribution 4.0 International License (http://creativecommons.org/licenses/by/4.0/), which permits unrestricted use, distribution, and reproduction in any medium, provided you give appropriate credit to the original author(s) and the source, provide a link to the Creative Commons license, and indicate if changes were made.
